# Book Reviews

**Published:** 1918-08

**Authors:** 


### BOOK REVIEW.

A Reference Handbook of the Medical Sciences, Third Edition, by Various Writers. Edited by Thomas Lathrop Stedman, M. D. In Eight Volumes. Illustrated by Chromolithographs and upwards of Six Thousand Engravings in the Text. Price, Cloth $7.00 per volume. Sold only by subscription. New York: William Wood and Company.

The completion of the third edition of this great work may justly be regarded as an event in medical publishing. This work, the first and the only complete medical encyclopedia in the English language, has enjoyed a long period of medical favor, the first edition having been completed in 1887. Its plan is that of the general encyclopedias, the various articles being presented alphabetically, thus permitting of ready reference, and it appeals to all classes of medical men, specialists as well as general practitioners and surgeons. There is no man, no matter how wide his experience or how great his medical learning, who cannot find here something to increase his knowledge or to help him in time of anxiety when worried by some unusual or perplexing case.

That the more than 3500 articles are well written and authoritative is shown by the list of nearly five hundred contributors, nearly all of them well known to the profession at large. Among these are: Alexander Lambert, Ph. B., M. D., New York, President Elect American Medical Association; Chas. C. Bass, M. D., New Orleans; Henry Dickson Bruns, M. D., New Orleans; William S. Carter, M. D., Galveston; George Dock, M. D., A. M., Sc. D., St. Louis; Isadore Dyer, Ph. B., M. D., New Orleans; Duncan Eve, A. M., M. D., Nashville; William Keiller, M D., F. R. C. S., Galveston; W. A. Pusey, A. M., M. D., Chicago; Edmond Souchon, New Orleans; Royal Whitman, M. D., New York.

The reviewer has through long experience learned to turn to this work whenever information on any medical subject is desired, and very seldom has he been disappointed in finding what he wanted. The present edition contains articles on many more topics than either of the pervious editions, as is but natural since the science of medicine is constantly expanding. The illustrations are excellently done, a large number of them being evidently new, and the printing and binding of the volumes are all that could be desired. It is difficult to conceive of a really useful medical library which has not this encyclopedia of medicine on its shelves.

